# Impact of the Arthritis Foundation’s Walk With Ease Program on Arthritis Symptoms in African Americans

**DOI:** 10.5888/pcd11.140147

**Published:** 2014-11-13

**Authors:** Brooke Wyatt, Chivon A. Mingo, Mary B. Waterman, Patience White, Rebecca J. Cleveland, Leigh F. Callahan

**Affiliations:** Author Affiliations: Brooke Wyatt, George Washington University School of Public Health, Public Health Department, The Arthritis Foundation, Washington, DC; Chivon A. Mingo, Georgia State University, Atlanta, Georgia; Mary B. Waterman, Patience White, Public Health Department, The Arthritis Foundation, Washington, DC; Rebecca J. Cleveland, Thurston Arthritis Research Center, University of North Carolina at Chapel Hill, Chapel Hill, North Carolina; Leigh F. Callahan, Thurston Arthritis Research Center, Cecil G. Sheps Center for Health Services Research, University of North Carolina at Chapel Hill, Chapel Hill, North Carolina.

## Abstract

**Introduction:**

Inadequate program design and lack of access to evidence-based programs are major barriers to the management of chronic diseases such as arthritis, particularly for African Americans. This study evaluates the effectiveness of the Arthritis Foundation’s Walk With Ease Program (WWE) in a subsample of African Americans who were part of a larger study that established evidence of the program’s efficacy.

**Methods:**

Participants were African Americans (N = 117) with self-reported arthritis who chose to participate in either a self-directed (n = 68) or group (n = 49) 6-week WWE program. Arthritis-related symptoms (ie, pain, fatigue, stiffness; measured using visual analog scales) were assessed at baseline, 6 weeks, and 1 year. Independent samples *t* tests were conducted to examine group differences (ie, self-directed vs group) in arthritis-related symptoms at baseline, and paired sample *t* tests were conducted to examine differences over time (ie, baseline to 6 weeks and baseline to 1 year) in symptoms. Satisfaction was examined by descriptive statistics.

**Results:**

Younger, more educated individuals chose the self-directed format (*P* < .001, *P* = .008; respectively). After the 6-week intervention, participants reported a decrease in pain (*P* < .001), fatigue (*P* = .002), and stiffness (*P* < .001). At 1 year, the decrease in pain (*P* = .04) and stiffness (*P* = .002) remained constant. Overall, participants were satisfied with both program formats.

**Conclusion:**

The individualized and group formats of the WWE program improved arthritis-related pain, fatigue, and stiffness in African Americans. Culturally appealing arthritis interventions ultimately may increase the use of existing arthritis interventions.

## Introduction

Arthritis, the leading cause of disability in the United States, affects almost 50 million Americans (1) and continues to be a growing public health concern. Arthritis, like most chronic conditions, disproportionately affects African Americans (2–4). Individuals with arthritis often experience pain, fatigue, stiffness, and activity limitations resulting in poor quality of health. African Americans experience these symptoms at a higher rate and with greater severity than whites (5–7). Therefore, identifying ways to minimize the negative impact of arthritis among African Americans is critical.

Evidence-based arthritis interventions (eg, Arthritis-Self Management Program [ASMP], Arthritis Foundation Aquatics Program [AFAP], Walk With Ease [WWE]) are effective in reducing arthritis-related symptoms such as pain, fatigue, and stiffness and are a useful treatment option for people with arthritis (8–13). However, most research on intervention programs for people with arthritis has been conducted predominantly among white populations with limited focus on African Americans (9,11,14). Few arthritis intervention studies that include African Americans examined preferences (15), effectiveness (16,17), cultural relevance, acceptability (16), satisfaction, and uptake specifically among this target population (10,15). Because of the paucity of research in this area, this study was undertaken to further the research by examining the effectiveness of the Arthritis Foundation’s Walk With Ease program among African Americans.

Specifically, our study is focused on a subset of the parent study population that was used to establish evidence of effectiveness for WWE in general (10). To date, no studies have focused on African Americans in this particular intervention program. Currently, WWE is delivered in 2 formats (self-directed or instructor-led group) (10). The primary objectives of this study were to 1) examine the effectiveness of the WWE program on improving the most common arthritis symptoms (pain, fatigue, and stiffness) in African Americans irrespective of delivery format, and 2) assess the target audience’s acceptability of and satisfaction with both the self-directed and instructor-led group WWE program. Although data from the parent study preclude the ability to examine effectiveness of WWE by delivery format in this population, our secondary objective is to examine differences in intervention delivery format selection across demographic variables and arthritis symptoms.

## Methods

The Arthritis Foundation’s WWE program, currently offered in 2 formats (ie, self-directed or instructor-led group), is a community-based 6-week program for adults with arthritis. The program focuses on setting goals, developing action plans, identifying motivational strategies, and building one’s confidence to increase physical activity as a way to minimize arthritis-related symptoms. Participants in the self-directed program are provided with the WWE workbook as a guide to navigate the 6-week program on their own. Participants in the instructor-led group program attend a class that meets 3 times a week for 1 hour, taught by lay leaders who have received a certification after attending a 1-day Arthritis Foundation Leader Training course.

Participants in the parent study included 462 community-dwelling adults recruited from senior centers, aging councils, public health departments, medical centers, rheumatology clinics, fitness/wellness centers, retirement communities, colleges and universities, churches, a service sorority, recreation centers, and various employers across urban and rural North Carolina counties (10). Eligible participants included individuals aged 18 years or older, self-reporting a diagnosis of arthritis, with no serious medical conditions (eg, uncontrolled hypertension or diabetes) or cognitive decline, and able to speak English. Participants chose either the self-directed or group program. Baseline assessments were collected starting in June 2008. Performance and self-reported outcomes were assessed at baseline and 6 weeks, and self-reported outcomes again at 1-year follow-up. The focus of this substudy is on the 117 participants who self-identified as African American and completed the self-reported assessments. The method used to select participants from the original WWE cohort for the purpose of this study is delineated (Figure). Additional details of the total sample have been reported elsewhere (10).

**Figure:**
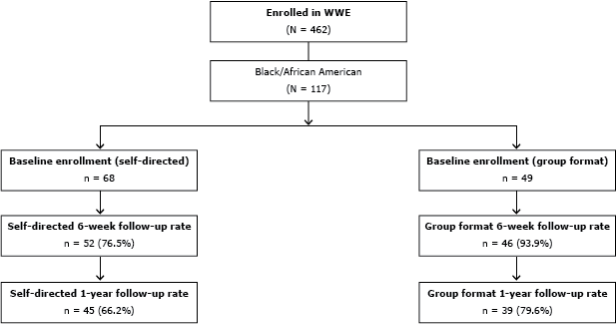
Walk With Ease (WWE) Participant Selection (African Americans)

The parent study was initially performed at 33 community sites throughout North Carolina, 20 of which had participants who reported being African American. Sites included senior centers, churches, community/health wellness centers, employers, and departments or councils on aging. To protect the privacy of participants where only 1 or 2 participants signed up, we do not provide detailed information by site. At baseline, each participant who provided written informed consent completed a self-report survey (on paper or computer-based), and a series of performance-based tests administered by a trained research team member was included in the study. Six-week follow-up assessments were conducted at each of the community sites, where participants completed the same initial tests and survey and a written satisfaction survey comprising closed and open-ended questions about their experience. One year after completion, participants were mailed surveys assessing their self-reported outcomes only, to evaluate long-term effects of the program. For the purposes of this study, we focus on self-report survey data only. All procedures for the parent study were approved by the University of North Carolina, Chapel Hill Biomedical Institutional Review Board, and by the Institutional Review Board of George Washington University for the substudy.

### Measures

**Demographics**. Demographic information included race, age, education, sex, body mass index (BMI), health status, and comorbid conditions. Race was assessed by asking participants, “What is your race/ethnicity?” Age was measured as a continuous variable by using participant self-reported date of birth and later stratified into 3 categories (<60 y, 60–74 y, ≥75 y). Education was assessed on the basis of the response to “What is the highest degree or level of school you have completed?” Responses were later dichotomized (having a high school education or less and having more than a high school education). BMI (kg/m^2^) was a continuous measure that was calculated by using self-reported height and weight and later dichotomized as less than 30 kg/m^2^ or 30 kg/m^2^ or more. Health status was assessed by asking participants to rate their health in general as excellent, good, fair, or poor (17). Participants were asked to report to each condition they had from a list of 16 common chronic conditions. Comorbid conditions represent the sum of all self-reported conditions not related to arthritis. To account for factors that may influence differences, demographic variables were included as covariates (18). All baseline and 6-week data were collected at the Assessment Center, a component of the National Institutes of Health Patient-Reported Outcomes Measurement Information System (PROMIS) initiative (19).

**Arthritis symptoms.** Pain, fatigue, and stiffness were measured by using visual analog scales (VAS) (20,21). VAS is a validated tool used to measure self-reported pain in participants and shows significant, reproducible findings across various longitudinal studies (22). Participants were asked to indicate a spot on a 100-mm line corresponding to their pain experience during the preceding 7 days. For each arthritis-related symptom (ie, pain, fatigue, and stiffness), the VAS is anchored with descriptors “no (symptom)” and “(symptom) as bad as it could be or is a major problem.” The VAS was then measured in millimeters from the left anchor to the point marked by the participant. Higher VAS scores (0–100) indicate more pain, fatigue, or stiffness. With pain, fatigue, and stiffness serving as the most common arthritis-related symptoms, these VAS variables were chosen to account for effectiveness of this substudy.

**Satisfaction.** Participants in both self-directed and group delivery formats were surveyed about their overall satisfaction with WWE. The survey assessed satisfaction and acceptability of information, tools, workbook, program presentation, value, or benefit. The survey questions were formatted differently depending on the type of program (ie, self-directed or group); however, answers were standardized for comparison of overall satisfaction. For example, in the group format, participants were asked: “Overall, to what extent are you satisfied with this program? (1 = Very well, 2 = Fairly well, 3 = A little, 4 = Not at all).” In the self-directed format, participants were asked, “Overall opinion of WWE program: I was satisfied with my experience doing the Walk With Ease program (1 = Strongly disagree, 2 = Disagree, 3 = Agree, 4 = Strongly agree).” Although the self-directed and group satisfaction survey results cannot be quantitatively compared, they are described in this study.

### Statistical analysis

To gauge the sensitivity of the statistical analyses used in this study, a power analysis was conducted on one of the main variables, pain, which was measured using the VAS. We found a minimal detectable difference (MDD) of 6.9 units with 80% power (n = 117; α = 0.05, standard deviation [SD], 26.4). This difference approaches the recommended VAS pain MDD range of 7 to 37 units and detects smaller differences as baseline pain scores decrease (23–25).

All analyses were conducted using SAS version 9.2. Descriptive statistics for demographic and outcome variables (ie, pain, fatigue, stiffness) were calculated separately for both the self-directed and group walking formats to provide a summary of the sample distribution at baseline. Differences in delivery formats were examined by using *t* tests and χ^2^ tests. Pearson correlations were conducted to assess if any of the demographic factors served as covariates when comparing the 2 program delivery formats.

We assessed the effectiveness of the intervention by comparing the arthritis symptom outcome scores (ie, pain, fatigue, and stiffness) at 6 weeks and at 1 year (independently) with baseline scores using a paired *t* test. Following the standard set forth in the parent study (10), effect sizes were calculated by using Cohen’s *d* (26) to determine if the change over time is considered meaningful. A change score resulting in an effect size of 0.1 to 0.3 indicates a modest effect, and a change score resulting in an effect size of 0.3 to 0.5 indicates a moderate effect (10). Analyses were restricted to the 98 African American participants who completed follow-up at 6 weeks (84%) and at 1-year follow-up (72%). However, no significant differences were found in demographics between people who dropped out of the program and people who continued the study. Descriptive statistics are presented to summarize the satisfaction of both the self-directed and group WWE program at 6 weeks and 1 year.

## Results

Of the 117 African American participants enrolled in the parent study at baseline, 68 were enrolled in the self-directed format and 49 were enrolled in the group format. Participants’ average age was 62; approximately 33% had a high school diploma or less. Most participants were female and reported being in excellent or very good health. More than 50% were classified as obese (Table 1).

**Table 1:** Participant Demographics and Differences Between Self-Directed and Group Format at Baseline for African Americans in Walk With Ease Study, North Carolina, 2008

Demographics	Self-Directed (n = 68)	Group (n = 49)	*P* Value^a^	All Baseline Participants (n = 117)^b^
Age, mean years (SD)	58.4 (12.5)	66.3 (8.3)	<.001	61.7 (11.6)
Education (≤high school, %)	23.5	46.9	.008	33.3
Female (%)	95.6	89.8	.22	93.2
BMI ≥30, (%)	55.9	57.1	.89	56.4
General health excellent/very good, (%)	26.9	20.4	.42	24.1
Comorbidities, mean (SD)	1.9 (1.0)	2.2 (1.3)	.17	2.1 (1.1)
**Arthritis symptoms^c^, median**				
Pain	31	30	.55	30
Fatigue	34	27	.48	32
Stiffness	40	42	.41	41

Abbreviations: SD, standard deviation; BMI, body mass index.

a Result of *t* test (continuous variables) or χ^2^ test (categorical variables) for assessing significance of differences between group and self-directed participants.

b Missing one observation for general health and 24 observations for comorbidities.

c Visual analog scale (VAS) measures (range, 0–100).

Significant differences were found in the age and education level of people selecting the self-directed versus the group format (Table 1). Individuals who selected the self-directed format were significantly younger and more educated than those who chose the group format (*P* < .001; *P* < .008, respectively). No significant differences were found in average number of comorbidities. However, a large number of participants in each format reported having high blood pressure (n = 70, 75%) and diabetes (n = 23, 25%) at baseline (data not shown). No significant group differences were found for BMI, health status, pain, fatigue, or stiffness.

Arthritis symptoms at baseline and 6-week follow-up were assessed to determine if there was improvement, irrespective of delivery format. A significant difference was found for all 3 arthritis symptoms, with the most significant changes occurring in pain and stiffness (effect size [ES] = 0.46 and 0.43, respectively) (Table 2). Participants reported less pain (ES = 0.31, *P* = .04) and stiffness (ES = 0.42, *P* = .002) from baseline to 1 year (Table 3). We found no significant difference for fatigue, despite a small difference in means of 4.7.

**Table 2:** Decreases in Arthritis Symptom Test Results From Baseline to 6-Week Follow-up Among African Americans in Walk With Ease Study, North Carolina, August*–*November 2008

Arthritis Symptoms^a^	N	Mean Decrease (95% CI)^b^	Effect Size	*P V*alue^c^
Pain	98	11.7 (7.5 to 15.8)	0.46	<.001
Fatigue	98	9.3 (4.9 to 13.6)	0.36	.002
Stiffness	98	12.2 (7.4 to 17.0)	0.43	<.001

Abbreviations: N, number of participants; CI, confidence interval.

a Visual analog scales (VAS) measures.

b Adjusted for age, sex, education, obesity, general health, and number of comorbidities.

c Result of *t* test for assessing significant differences between mean baseline and 6-week follow-up VAS scores.

**Table 3:** Decreases in Arthritis Symptom Test Results From Baseline to 1-Year Follow-up Among African Americans in Walk With Ease Study, North Carolina, August–November 2009

Arthritis Symptoms^a^	N	Mean Decrease (95% CI)^b^	Effect Size	*P* Value^c^
Pain	86	8.7 (2.9 to 14.6)	0.31	.04
Fatigue	84	4.7 (−0.2 to 9.6)	0.19	.31
Stiffness	84	11.6 (6.5 to 16.8)	0.42	.002

Abbreviations: N, number of participants; CI, confidence interval.

a Visual analog scales (VAS) measures.

b Adjusted for age, sex, education, obesity, general health, and number of comorbidities.

c Result of *t* test for assessing significant differences between mean baseline and 6-week follow-up VAS scores.

Of the 71% of African American participants (n = 48) who completed the satisfaction survey after participating in the self-directed format at 6-week follow-up, 92% (n = 44) agreed or strongly agreed that they were satisfied with the overall WWE program (data not shown). In surveying the group format participants at 1-year follow-up, data were collected anonymously; therefore, satisfaction specifically for African Americans could not be determined. However, overall, participants enrolled in the group format were indeed satisfied. Of the 109 group participants who completed follow-up surveys in the original study, 100% reported that they would recommend the WWE program to a friend. Nearly all of the group format participants also reported that their instructors kept them interested in the program at a fairly or very well level (99%), that they were satisfied with the way the instructor presented the topics (100%), and that the program fulfilled their expectations (99%).

## Discussion

This study evaluated the effectiveness of WWE in African Americans with arthritis, who are disadvantaged in the impact and prevalence of the most common symptoms associated with arthritis (4,5). Results indicate that both pain and stiffness improved, with stiffness improving significantly at both 6-weeks and 1-year. Moreover, fatigue was significantly reduced at 6 weeks. However, the difference in fatigue from baseline to 1 year was not significant and fell below our indicated range of a meaningful change. Although findings were similar, the effect sizes found in our study examining only African Americans were slightly higher than those presented in the parent study, suggesting a greater impact. Findings in the parent study were presented by program delivery format whereas our findings are irrespective of delivery format. Because knowledge about the effectiveness of arthritis behavioral interventions for African Americans is limited (9,10), our results are promising. They contribute to the public health literature by providing a foundation for future studies focused on designing and examining the effectiveness of culturally appealing interventions that could aid in mitigating existing arthritis-related disparities.

WWE not only reduced arthritis-related symptoms but was also perceived as acceptable by African American participants. Specifically, most participants in the self-directed program reported being satisfied and having completed the program with lessons learned. Because differences in demographics were negligible by program format, it is plausible that African Americans’ overall satisfaction with the self-directed format is similar to their overall satisfaction with the group format. Previous research has found that African Americans with arthritis respond favorably to programs beneficial for their condition that could be delivered to them independently or in a group setting (16). Therefore, offering a program with optional delivery formats may be a way to engage an African American population that has consistently been underrepresented in interventions of this type. However, having the option of an independent versus group program may not affect acceptability, satisfaction, and participation among this underrepresented population. Other structure and delivery factors (eg, encouraging participation of others similar in race, sex, and age, including instructor of same race) may act as interconnecting facilitators or barriers to participation in arthritis behavioral interventions, influencing acceptability and satisfaction. Therefore, future research should consider additional delivery and structure components that may improve the appeal, cultural relevance, acceptability, and satisfaction of behavioral interventions among this underrepresented population.

Findings from this study have implications specifically for African Americans. Having a physical activity program that minimizes disease-related symptoms and is deemed acceptable among this target population provides a foundation for future research. Not only do African Americans have worse arthritis outcomes than other races/ethnicities (5,6), they are also overrepresented in being diagnosed with multiple chronic conditions (2,3) and underrepresented in meeting physical activity recommendations (27,28). Reducing disparities in chronic disease, specifically in arthritis, and in physical activity are national efforts included in Healthy People 2020 (29). Walking reduces the risk of various chronic conditions and minimizes symptoms (29). Increasing African Americans’ participation in physical activity programs that have multiple health benefits could decrease the chronic disease health disparities. Future research should further examine the effectiveness of programs like WWE and identify innovative ways to increase their public health impact.

Our study has limitations. Although our study was adequately powered to detect the recommended MDD for our primary outcomes (pain, stiffness, and fatigue), the small sample size prevented additional analyses that could determine differences in symptoms according to delivery format. However, the findings still provide novel information about the effectiveness of WWE among African Americans. Using 2 different surveys and following 2 different methodologies may have limited our ability to determine any differences in satisfaction according to format. Because we used participant self-report to measure arthritis symptoms, responses may have been biased due to social desirability. However, the scale used for this study has been used in similar research as a valid measure for pain, fatigue, and stiffness. In addition, future research examining the effectiveness of a behavioral arthritis intervention should include a control group to provide stronger evidence that the effects found are due to participation in the intervention. Notably, this sample of African Americans was highly educated, predominantly female, and reportedly in good health. Demographics of this type limit the external validity of our findings. Lastly, a mixed-method (quantitative and qualitative) design may have been a stronger methodological approach for assessing satisfaction and acceptability.

Despite these limitations, our study provides practical lessons. For example, we used targeted strategies (eg, recruiting from senior centers in neighborhoods with a higher percentage of African Americans) in an effort to overcome barriers to recruiting and retaining African Americans. Recruitment efforts resulted in 25% of the WWE parent study population being African American (10); this percentage exceeds the national average (14.2% identify as African American) (3). More than 70% of the participants remained in the study over time. Relationships between obesity, other chronic conditions, and behavioral walking interventions should be examined through further research.

From a public health perspective, a community-based walking program for African Americans with arthritis has the potential to increase the currently limited culturally sensitive self-management opportunities now offered (10). Our findings provide the foundation for beginning to address arthritis-related health disparities through culturally sensitive, acceptable effective behavioral interventions.
